# Prevalence and molecular identification of gastrointestinal nematodes in Qinghai‐Tibetan Plateau of China

**DOI:** 10.1002/vms3.674

**Published:** 2023-10-26

**Authors:** Sitong Ai, Zhichao Zhang, Jinghan Wang, Xiaoqi Wang, Cheng Liu, Ziyuan Duan

**Affiliations:** ^1^ Institute of Genetics and Developmental Biology Chinese Academy of Sciences Beijing China; ^2^ Department of Animal Husbandry and Veterinary Medicine College of Agriculture Eastern Liaoning University Dandong China

**Keywords:** gastrointestinal nematodes (GINs), livestock, molecular identification, prevalence, Qinghai‐Tibetan Plateau (QTP)

## Abstract

**Background:**

Gastrointestinal nematodes (GINs) have seriously affected the production and earnings of animal husbandry in various countries, while some species of GINs infect humans. At present, little is known about the species and prevalence of GINs in Qinghai‐Tibetan Plateau (QTP).

**Methods:**

In this study, 528 fresh faecal samples were collected from typical areas in different altitudes with seven species of livestock in Qinghai, Tibet, Gansu and Yunnan Provinces. ITS‐2 rRNA gene of nematodes was employed to detect by PCR and sequencing analysis. Phylogenetic analysis of related sequences was performed using MEGA 6.0 software.

**Results:**

The overall prevalence of GINs was 80.3% with 20 species of GINs detected, while *Teladorsagia circumcincta* was the dominant one, and four of which were zoonotic species such as *Trichostrongylus colubriformis*, *Trichostrongylus axei*, *Trichostrongylus vitrinus* and *Oesophagostomum stephanostomum*.

**Conclusion:**

The study provided panoptic information on the prevalence and species diversity of GINs in QTP area, which is useful and valuable for reference of measure formulation in livestock husbandry and public health concerns. The parasites of *T. circumcincta*, *Cylicocyclus nassatus*, *Strongylus edentatus*, *Cylicostephanus longibursatus*, *Telephlebia brevicauda*, *Cyathostomum catinatum*, *Mecistocirrus digitatus*, *Cooperia punctata*, *Cylicodontophorus bicoronatus*, *Nematodirus oiratianus* and *Oesophagostomum asperum* were firstly reported the presence in QTP area. The study also showed that horse could be infected by *T. circumcincta*, goat could be infected by *C. nassatus*, cattle could be infected by *S. edentatus* and *C. bicoronatus*,and *O. stephanostomum* could infect yak, cattle and Mongolian sheep in worldwide. Nevertheless, more investigations are needed, such as microscopic examination, to accurately determine the species in the region.

## Graphical abstract

1. Provided panoptic information on the prevalence and species diversity of GINs in QTP of China.

2. Some gastrointestinal nematodes that have not been reported in this area were detected.

3. *O. stephanostomum*, *T. circumcincta*, *C. nassatus*, *S. edentatus* and *C. bicoronatus* can transmit cross‐species.

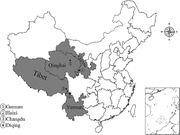

## 1 INTRODUCTION

Gastrointestinal nematodes (GINs), as a common parasite are widely distributed in the worldwide (Saidi et al., 2020), giving rise to reduced production and economic losses in husbandry (Barghandan et al., 2020), are a major constraint to the survival and productivity of animals (Yuan et al., 2019). There have been many reports on the host of domestic or wild animals infected with GINs, such as sheep (Ploeger & Everts, 2018), European bison (Demiaszkiewicz et al., 2020) and chimpanzees (McLennan et al., 2017). Some species of GINs can also infect humans through ingested infective‐stage larvae (Gholami et al., 2015) and cause zoonotic infections due to the wide range of human activities, which pose a challenge to public health concerns.

The Qinghai‐Tibetan Plateau (QTP), with its average elevation over 4000 m, low annual temperature and oxygen content, and changeable climate, is the highest and largest plateau in China (Tang et al., 2018; Zhang et al., 2019). As the source of three rivers (the Yangtze River, Yellow River and Mekong River) (Jian et al., 2018), there is a large proportion of livestock husbandry in economy, with yak and Tibetan sheep as the primary livestock breeds. Due to the primitive breeding manners in the area, the livestock management is mainly family‐running with extensive husbandry in small group rearing and lower productivity (J.‐Z. Liu et al., 2016). When grazing in steppe of the Plateau, the animals may be infected with water and grass contaminated with eggs of nematodes. Once infected, these animals continuously excrete the faeces containing the eggs due to the failure of timely intervention, resulting in mutual infection within the same herd, which further aggravates the pollution of grassland and water, and poses a threat to the health of residents and livestock in downstream.

Although there are a variety of livestock and wild animals living in QTP area, few investigations have been carried out on the species and prevalence of GINs in the region. In this study, we conducted an epidemiological investigation of GINs in seven species of asymptomatic domestic animals from the QTP in order to provide references for animal husbandry and public health.

## 2 MATERIALS AND METHODS

### 2.1 Sample collection

**FIGURE 1:**
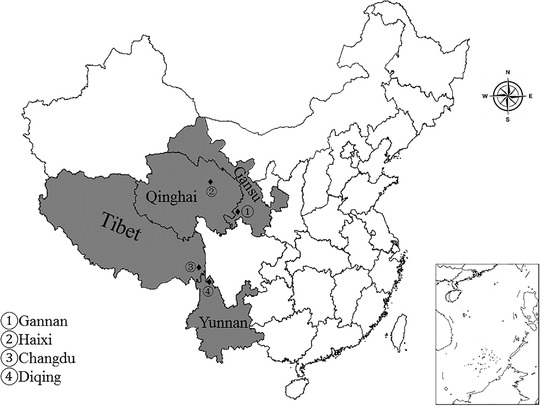
The location of the sampling site selected for this study. ♦ Sampling site

A total of 528 fresh faecal samples were collected from seven species of domestic animals without obvious clinical symptoms of horse (*n* = 32), camel (*n* = 40), yak (*n* = 145), cattle (*n* = 57), goat (*n* = 94), Tibetan sheep (*n* = 121) and Mongolian sheep (*n* = 39) during May to June in last year. These samples were collected from four sites in the QTP, such as Haixi Mongolian and Tibetan autonomous prefecture in Qinghai Province (average altitude 3000 m), Changdu city in Tibet Autonomous Region (average altitude 4000 m), Gannan Tibetan autonomous prefecture in Gansu Province (average altitude 3100 m) and Diqing Tibetan autonomous prefecture in Yunnan Province (average altitude 3300 m) (Figure 1). The fresh faeces samples were collected quickly as soon as large animal excreted and placed into sterile tubes immediately. For small ruminants, the samples were directly acquired from the rectum of sheep or goat by hand covered with a sterile glove then placed into sterile tubes. Each of the tube was given a unique number, properly frozen with ice packs and immediately sent to the laboratory for further analysis.

### 2.2 DNA extraction and PCR amplification

Two hundred milligrams of faecal samples were taken out from the sterile tube to extract the genomic DNA by using TIANamp Stool DNA Kit (TIANGEN Biotech, Beijing, China) following the manufacturer's instruction. One of the steps was modified with lysis temperature increased from 70 to 95℃ to obtain high quality and yield of DNA. The extracted DNA was divided into three tubes, one of which was used for PCR amplification, and the other two tubes were stored at −80℃ for further validation. A pair of primers from checked literature and with validation in pre‐experiment (forward primer: TAGCTTCAGCGATGGATCGGT; reverse primer: CTTTTCCTCCGCTAAATGATATGC) which could amplify the products of approximate 500 base pairs were used for targeting the ITS‐2 rRNA gene to detect GINs as described previously (Bisset et al., 2014). The PCR amplifications was performed in a 25 μl reaction system with 12.5 μl of 2× Taq DNA polymerase Master Mix (Vazyme Biotech, Nanjing, China), 1 μl of 10 μM of each primer, 2 μl of DNA template and 8.5 μl of double distilled water. The PCR programme was performed as follows: the initial denaturation at 95°C for 8 min, followed by 35 cycles at 94°C for 30 s, 55°C for 30 s and 72°C for 40 s, then extension at 72°C for 7 min. The PCR products were identified by 2% agarose gel with EB stained electrophoresis.

### 2.3 Sequencing and phylogenetic analysis

The obtained positive PCR products were sent to BGI (Beijing) for sequencing. All received sequences were searched by BLAST first, and the nematodes species corresponding to the matched homologous sequence (E‐value = 0, identity > 96%) was obtained and identified as the species infected by the sample of the collected animal.

The representative nucleotide sequences acquired in the study have been submitted to the GenBank database under the accession numbers MT193646 and MT193665. Phylogenetic tree of related nematodes was constructed using the neighbour‐joining algorithm and Kimura 2‐parameter model in MEGA 6.0 software, and the robustness of the tree topology was assessed using 1000 bootstrap replicates (Zhang et al., 2019).

### 2.4 Data analysis

The data were analyzed by the chi‐squared test using SPSS 17.0 software to calculate the infection rates of GINs in different animals, elevations and regions. *p*‐Values < 0.05 were considered a significant difference criterion.

## 3 RESULTS

### 3.1 Prevalence and distribution

The total infection rate of GINs against seven species of livestock was 80.3% (424/528) (Table 1), which ranged from 75.0% to 89.9% for different regions and altitudes (Table 2), and 56.4% to 98.9% for different animals. In the view of four distribution regions, Tibet had the highest infection rate 89.9% (71/79), followed by 89.7% (78/87) in Yunnan, 76.5% (182/283) in Qinghai and 75.0% (93/124) in Gansu. Among different species of animals, goats had a highest infection rate of 98.9% (93/94), followed by camels (37/40, 92.5%), Tibetan sheep (104/121, 86.0%), yaks (110/145, 75.9%), horses (24/32, 75.0%), cattle (34/57, 59.6%) and Mongolian sheep (22/39, 56.4%).

**TABLE 1:** Detection and species distribution of gastrointestinal nematodes (GINs) in different kinds of livestock in the Qinghai‐Tibetan Plateau (QTP) of China

Animal	Location	Number of samples	Number of positives (%)	Species (No.)
Horse	Qinghai, Haixi	32	24 (75.0)	*Cylicocyclus nassatus* (8) *Strongylus edentatus* (6) *Cylicostephanus longibursatus* (5) *Teladorsagia circumcincta* (2) *Triodontophorus brevicauda* (2) *Cyathostomum catinatum* (1)
Camel	Qinghai, Haixi	40	37 (92.5)	*Trichostrongylus colubriformis* (35) *Trichostrongylus axei* (1) *Trichostrongylus vitrinus* (1)
Yak	Qinghai, Haixi; Tibet, Changdu; Gansu, Gannan; Yunnan, Diqing	145	110 (75.9)	*Cooperia oncophora* (57) *Trichostrongylus axei* (20) *Oesophagostomum stephanostomum* (14) *Ostertagia ostertagi* (9) *Trichostrongylus colubriformis* (3) *Teladorsagia circumcincta* (3) *Trichostrongylus vitrinus* (3) *Chabertia ovina* (1)
Cattle	Qinghai, Haixi; Yunnan, Diqing	57	34 (59.6)	*Trichostrongylus axei* (12) *Teladorsagia circumcincta* (6) *Mecistocirrus digitatus* (5) *Oesophagostomum stephanostomum* (4) *Cooperia oncophora* (3) *Strongylus edentatus* (1) *Marshallagia marshalli* (1) *Cooperia punctata* (1) *Cylicodontophorus bicoronatus* (1)
Tibetan sheep	Qinghai, Haixi; Tibet, Changdu; Gansu, Gannan	121	104 (86.0)	*Teladorsagia circumcincta* (55) *Marshallagia marshalli* (36) *Trichostrongylus axei* (7) *Oesophagostomum asperum* (3) *Haemonchus contortus* (2) *Trichostrongylus colubriformis* (1)
Goat	Qinghai, Haixi; Yunnan, Diqing; Gansu, Gannan	94	93 (98.9)	*Teladorsagia circumcincta* (39) *Marshallagia marshalli* (26) *Haemonchus contortus* (17) *Nematodirus oiratianus* (5) *Trichostrongylus axei* (4) *Trichostrongylus colubriformis* (1) *Cylicocyclus nassatus* (1)
Mongolian sheep	Qinghai, Haixi	39	22 (56.4)	*Teladorsagia circumcincta* (16) *Oesophagostomum stephanostomum* (5) *Trichostrongylus vitrinus* (1)
Total		528	424 (80.3)	*Teladorsagia circumcincta* (121) *Marshallagia marshalli* (63) *Cooperia oncophora* (60) *Trichostrongylus axei* (44) *Trichostrongylus colubriformis* (40) *Oesophagostomum stephanostomum* (23) *Haemonchus contortus* (19) *Ostertagia ostertagi* (9) *Cylicocyclus nassatus* (9) *Strongylus edentatus* (7) *Cylicostephanus longibursatus* (5) *Mecistocirrus digitatus* (5) *Nematodirus oiratianus* (5) *Trichostrongylus vitrinus* (5) *Oesophagostomum asperum* (3) *Triodontophorus brevicauda* (2) *Chabertia ovina* (1) *Cooperia punctata* (1) *Cyathostomum catinatum* (1) *Cylicodontophorus bicoronatus* (1)

**TABLE 2:** Prevalence of gastrointestinal nematodes (GINs) in different areas and altitudes in Qinghai‐Tibetan Plateau (QTP) of China

Region	Number of samples	Number of positives	Prevalence (%)	Average altitude(m)	*p‐*Value
Gansu	124	93	75.0^A^	3100	0.003
Qinghai	238	182	76.5^AB^	3000	
Tibet	79	71	89.9^BC^	4000	
Yunnan	87	78	89.7^C^	3300	

If same letter has been used between any two groups, the difference is not significant; if different letters are used, the difference is significant.

### 3.2 Molecular identification

Twenty nematodes species were detected and identified by sequence analysis. Among them, the parasites *Teladorsagia circumcincta* was the dominant one with a total infection rate of 22.9% (121/528), which infected six species of livestock: horse, yak, cattle, Tibetan sheep, goat and Mongolian sheep. Four species of zoonotic nematodes in six species of livestock other than horses were identified, which was *Trichostrongylus axei* with prevalence rates 8.3% (*n* = 44, 44/528), *Trichostrongylus colubriformis* with 7.6% (*n* = 40, 40/528), *Trichostrongylus vitrinus* with 0.9% (*n* = 5, 5/528) and *Oesophagostomum stephanostomum* with 4.4% (*n* = 23, 23/528), and all positive samples detected in camels were *Trichostrongylus* spp. parasites. Besides *T. circumcincta* and *Marshallagia marshalli* (*n* = 63), parasites commonly found in ruminants, *Cooperia oncophora* (*n* = 60), were detected from cattle and yak. Yet, six species of parasites have few numbers identified in infection: *Cyathostomum catinatum* (*n* = 1) and *Triodontophorus brevicauda* (*n* = 2) detected from horse, *Cooperia punctata* (*n* = 1) and *Cylicodontophorus bicoronatus* (*n* = 1) detected from cattle, *Chabertia ovina* (*n* = 1) detected from yak and *Oesophagostomum asperum* (*n* = 3) detected from Tibetan sheep.

### 3.3 Phylogenetic analysis

**FIGURE 2:**
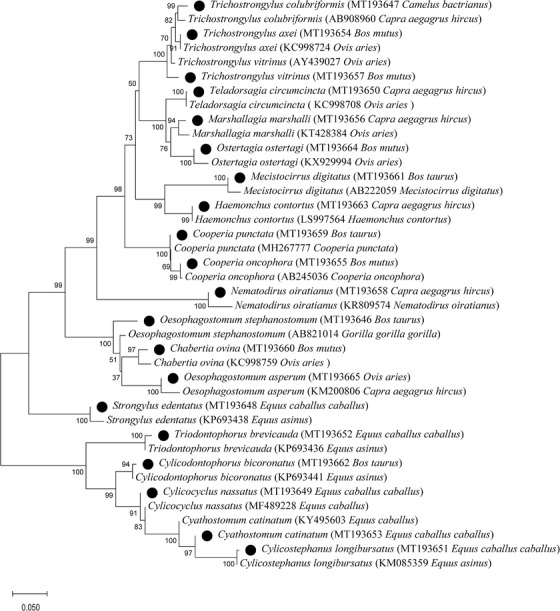
The phylogenetic relationship of the gastrointestinal nematodes (GINs) species detected in this study and those of related nematodes. Bootstrap values > 50% calculated from 1000 replicates are shown at the nodes. The black filled circles represent nematode species identified in the present study

The phylogenetic relationship of the GINs species was established, and their reference strains were acquired form Nucleotide Database in NCBI. All of the species of GINs identified and the original reference strains were clustered into a same clade, and the clustering results of the study were consistent with the literature (Sun et al., 2020) The two strains between the evolutionary relationships was also close (Figure 2), indicating that the results of our identification of GINs were credible.

## 4 DISCUSSION

GINs are the most common and economically destructive parasite in ruminants (El‐Alfy et al., 2019). Because of their diversity and different susceptibility to anthelmintic (T. Han, Wang, et al., 2017; Rashid et al., 2018), it is important to identify their species, hosts and infection sites to clarify which animals can be easily infected in specific areas.

As known, the eggs of different species of GINs are very similar in size and shape. In addition, the identification for third stage of infective larvae requires the assistance of experienced specialist, which makes the job prone to error and challenging (El‐Alfy et al., 2019; Redman et al., 2019); therefore, they are difficult to identify from each other. The molecular methods have the characteristics of rapidity and accuracy, which have great advantage for specific identification for different species of nematodes (Callaghan & Beh, 1994; Roeber et al., 2011). Former investigations on GINs in QTP were mostly on morphological ways (Cao et al., 2020; J.‐Z. Liu et al., 2016), few with specific molecular identification. In this study, the ITS‐2 sequence was used to identify the species of GINs, which has been proved suitable and effective (Bisset et al., 2014; Dallas et al., 2000; Santos et al., 2020; Tan et al., 2018).

Before the present study, there were a few reports on GINs infection among animals limited in one district or single province of the QTP area. For example, the infection of GINs on yaks, cattle, sheep, goats, horses, donkeys, Tibetan pigs and other domestic animals in Tibet district with 63.8% (Wu et al., 2017), which is lower than the infection rate of 80.3% detected in this study, and the infection in wild female Tibetan antelope in Qinghai Province with 93% (Cao et al., 2020), which is similar to 92.5% infection rate of camels measured in this study, and little related with GINs infection against all species of livestock in large areas of QTP region. Hence, we sampled whole typical area in different elevation distributed with domestic animals in the QTP and detected 20 species of GINs from the faeces of seven species of livestock with 80.3% infection rate. Further, we also found that the parasites of *T. circumcincta* were the dominant species (28.5%, 121/424) in this region, which is common in sheep and goats in other areas (Costa‐Junior et al., 2021; Hrabok et al., 2006) and also detected in large animals such as cattle, yaks and horses in this study. Up to now, there was a report on *T. circumcincta*‐infected cattle, which was a detection of antibodies against the parasite in cattle faeces and serum (no microscopic examination done) (Cooke et al., 2019). One document reported the isolation of the parasite from the small intestine of yaks (L. Han, Zhou, et al., 2017) but no report that the infection case in horses. Our study demonstrated that *T. circumcincta* could not only infect cattle and yaks but may infect horses, and then expended the host range of this parasite.

Outside of QTP, there were some reports on livestock infection with GINs in other areas of China (Bhuiyan et al., 2017; T. Han, Wang, et al., 2017; Lv et al., 2016; Shen et al., 2019; Yuan et al., 2019), such as the sheep in inner Mongolian with 79.2% infection rate (T. Han, Wang, et al., 2017), which is consistent with the 78.8% (126/160) of the sheep (Tibetan and Mongolian sheep) in QTP identified in this study. Combining our result and others in the recent study, the infection of GINs is widely distributed in China, indicating the severe situation that animal husbandry faced both in domestic livestock and wild animals.

From a previous study, it was widely believed that *T. axei*, *T. colubriformis, T. vitrinus* and *O. stephanostomum* could infect humans (Bundy et al., 1985; Cibot et al., 2015; Elseadawy et al., 2021; Ghadirian & Arfaa, 1975; Phosuk et al., 2013) as zoonotic parasites, and there were already documents showing the infection with *Trichostrongylus* spp. in Henan (0.13%), Jiangsu (0.07%) and Jilin (0.06%) in local residents (Guan et al., 2015; Lee et al., 2017; Wei‐Qi et al., 2017). Except for horses, these four species of zoonotic nematodes were all detected in six species of livestock in the QTP. Although there have been many reports on *O. stephanostomum* infecting animals and human in other countries (Cibot et al., 2015; Gasser et al., 1999; Krief et al., 2008; Terio et al., 2018), there is no case about the parasite infecting human in China at present. The infection rate of *T. axei* and *T. colubriformis* on Tibetan sheep detected in this study was 5.8% and 0.8%, respectively. Considering the similar investigation in sheep conducted in Heilongjiang Province with the infection rate of 10.4% and 36.8% (C. R. Wang et al., 2006), which was much higher than our results, indicating that the parasites of *T. axei* and *T. colubriformis* might be more suitable to live in regions at lower elevations than at higher ones while the management level in the former would not be much lower than in the QTP area. Till now, there was only one case about the infection of *O. stephanostomum* in China reported in Tibetan pigs (Li et al., 2017). This study identified that *O. stephanostomum* infected yaks, cattle and Mongolian sheep. Besides, the infection status on parasites of *Trichostrongylus* spp. evaluated in healthy yak in Gansu Province previously (Li et al., 2018) is the same as what we detected, suggesting that nomads in this region need to be vigilant for potential infection.

In addition, there were other four species of nematodes detected in the study, such as *T. circumcincta*, *Marshallagia marshalli*, *Haemonchus contortus* and *Ostertagia ostertagi*, considered as zoonotic potential as well. Looking through literatures, there have been two reports on human infection with these four parasites and the both occurred in Iran (Ashrafi et al., 2020; Ghadirian & Arfaa, 1973). Evidently, the scope and severity of zoonosis caused by these four parasites need to be further studied and determined. The nematodes of *Marshallagia* spp., *Haemonchus* spp. and *Ostertagia* spp. usually live in the abomasum and small intestine of ruminants such as camels, cattle and sheep (Besier et al., 2016; Lichtenfels et al., 1988). In this study, we also detected the *M. marshalli* in cattle, Tibetan sheep and goats, *H. contortus* in Tibetan sheep and goats and *O. ostertagi* in yaks. These results were consistent with a few investigations on domestic animals, such as *M. marshalli* infection of yaks (Sun et al., 2018) and wild animals, such as *M. marshalli* infection of Tibetan antelope and Przewalski's Gazelle in the QTP region (Cao et al., 2020; Sun et al., 2018; Y. Wang et al., 2016). As for *H. contortus* and *O. ostertagi*, both parasites have been widely reported their infections in ruminant livestock such as goats, yaks and sheep (including Tibetan sheep) in the QTP (Khan et al., 2019; Z. Liu et al., 2018). Another nematode infected yaks and cattle with relatively large amounts (n = 60) was *C. oncophora*, which is frequently parasitic in the small intestine of ruminants (Sun et al., 2020) and has been identified in yaks before in the QTP region (L. Han, Zhou, et al., 2017).

Beyond the above‐mentioned parasites, we have also found 11 species of nematodes in the QTP area but with a few infection numbers less than 10 individuals. Except for *C. ovina* detected in the goats, Tibetan sheep and yaks which had been reported several times in literatures infected yaks and Tibetan sheep in the QTP (Xu et al., 2016; L. Zhao et al., 2015). The rest of the 10 parasites, either some nematodes identified in the QTP for the first time, such as *Cylicostephanus longibursatus* (*n* = 5), *Triodontophorus brevicauda* (*n* = 2) and *C. catinatum* (*n* = 1) which are commonly found in equine animals (Gao et al., 2017; Tzelos et al., 2020) and detected in horse in this study, *Mecistocirrus digitatus*, *C. punctata* living in the abomasum and small intestine of ruminants (Paguem et al., 2020; von Son‐de Fernex et al., 2014) and also detected in cattle in the study. *Nematodirus oiratianus* and *Oesophagostomum asperum* commonly had been found in ruminants in other countries (Santos et al., 2020; G.‐H. Zhao et al., 2013, 2014) also detected in the QTP in the study. Some nematodes not only found in the QTP area but also out of original host and may have a wide range of infection. *Cylicocyclus nassatus* (*n *= 9) and *Strongylus edentatus* (*n* = 7) infected horses out of China (Fabiani et al., 2016; Kooyman et al., 2016; McClure et al., 1994; Studzinska et al., 2012; van Doorn et al., 2014) but infect goats and cattle in the study as well. *Cylicodontophorus bicoronatus* usually live in the colon and cecum of equine animals (Silva et al., 1999) but detected in cattle in the QTP area. The latter three parasites were found all beyond original hosts and may have a wide range to infect.

In the process of this study, we were surprised to see a number of parasites normally parasitic to ruminants were detected in horse, as well as normally parasitic to equine animals but found in ruminant faeces. Therefore, we re‐examined every process of sample collection, DNA extraction, PCR amplification, sequencing and alignment, reperformed the above experiments on the samples. Once again, the results above were confirmed, showing there was no fault such as sample and PCR contamination or the label confused. The reason of the results might be one possible way due to cross‐species transmission and mixed infection of these parasites caused by the livestock overlap in raising plot at night in this area, and hence more investigation would be needed in the area, such as microscopic examination. Because of the serious harm to humans and impeding to livestock production and livestock, more investigations and studies for GINs are needed to accurately determine the species, pathogenicity, distribution and prevalence at different altitudes and regions in the QTP.

## 5 CONCLUSION

This study included whole typical area in different elevation distributed with domestic animals and provided useful and valuable information on the prevalence and species diversity of GINs in QTP of China. A total of 20 species of GINs were detected and identified from seven species of domestic animals, four of which were zoonotic species, such as *T. axei*, *T. colubriformis*, *T. vitrinus* and *O. stephanostomum*. Some parasites detected the presence in QTP area for the first time, such as *T. circumcincta*, *C. nassatus*, *S. edentatus*, *C. longibursatus*, *T. brevicauda*, *C. catinatum*, *M. digitatus*, *C. punctata*, *C. bicoronatus*, *N. oiratianus* and *O. asperum*. To our knowledge, this is the first report identifying the presence of *O. stephanostomum* in yak, cattle and Mongolian sheep, *T. circumcincta* in horse, *C. nassatus* in goat, *S. edentatus* and *C. bicoronatus* in cattle worldwide, suggesting that *O. stephanostomum*, *T. circumcincta*, *C. nassatus*, *S. edentatus* and *C. bicoronatus* may have a wide range of hosts. The results indicated the severe situation that animal husbandry faced both in domestic livestock and wild animals.

## CONFLICT OF INTEREST

The authors declare no conflict of interest.

## ETHICS STATEMENT

All faeces are collected with the consent of the livestock owners. THE whole process of collecting stool samples is in accordance with the Guidance of Experimental Animal Welfare and Ethical Treatment issued by the Ministry of Science and Technology of China.

## AUTHOR CONTRIBUTIONS

Sitong Ai conducted the laboratory experiments and wrote the first draft. Zhichao Zhang, Xiaoqi Wang and Cheng Liu made contributions to the samples collection and processing. Jinghan Wang and Ziyuan Duan conceived and designed the experiment and revised the manuscript. All authors have read and approved the final manuscript.

## Data Availability

The datasets generated during the current study are not publicly available since our laboratory needs to do further research on these parasites but are available from the corresponding author (zyduan@genetics.ac.cn) on reasonable request.
